# Association of first trimester serum uric acid levels gestational diabetes mellitus development

**DOI:** 10.4274/tjod.69376

**Published:** 2016-06-15

**Authors:** Seda Şahin Aker, Tuncay Yüce, Erkan Kalafat, Murat Seval, Feride Söylemez

**Affiliations:** 1 Dr. Sami Ulus Maternity and Children Training and Research Hospital, Clinic of Obstetrics and Gynecology, Ankara, Turkey; 2 Ankara University Faculty of Medicine, Department of Obstetrics and Gynecology, Ankara, Turkey

**Keywords:** Hyperuricemia, risk assessment, gestational diabetes, screening

## Abstract

**Objective:**

To investigate the association of first trimester serum uric acid levels with the development of gestational diabetes mellitus (GDM) in low-risk pregnant women.

**Materials and Methods:**

In this retrospective data analysis, the results of pregnant women who completed both first trimester biochemical panel and two-step GDM screening were compared with an age-, body mass index, and gestational age-matched control group. The women were grouped as either GDM or impaired glucose tolerance (IGT) according to 100-g oral glucose challenge results. Uric acid levels were compared between the groups and diagnostic utility was tested with receiver-operating characteristics curves.

**Results:**

Sixty-six women in GDM group and 358 women in the IGT group were compared against 202 healthy pregnant women. The groups did not differ significantly in terms of parity, pre-gestational body mass index and gestational age. Serum samples for uric acid levels were obtained. The mean serum uric acid levels were significantly higher in the GDM and IGT groups (5.95 mg/dL (±0.97 mg/dL) and 4.76 mg/dL (±1.51 mg/dL), respectively) compared with the control group (3.76 mg/dL (±1.07 mg/dL) (p<0.001). The area under the curve for uric acid levels was 0.92 (95% confidence interval 0.88-0.95) for diagnosis of GDM. At a diagnostic threshold of 3.95 mg/dL, uric acid levels predicted development of GDM with 60% specificity and 100% sensitivity.

**Conclusion:**

First trimester serum uric acid has a linear association with the development of GDM and IGT.

## INTRODUCTION

Gestational diabetes mellitus (GDM) is a relatively common disorder of pregnancy. The prevalence of GDM ranges from 1 to 6% depending on the studied population^(1,2)^. Large population-based studies are lacking in Turkey but some available data from cohort studies suggest that the prevalence of GDM ranges between 4-10% in Turkey^(3,4,5)^. Prediction and diagnosis of GDM is important for ongoing pregnancy and has important implications for subsequent health of the mother. GDM is considered a significant risk factor for subsequent development of type II diabetes and is associated with a poorer cardiovascular risk profile compared with women without GDM^(6,7)^.

The method of screening (one-step versus two-step), application of screening (broad versus risk-dependent), and diagnostic criteria of GDM have been the subject to debate. Risk- dependent screening is being abandoned world-wide after the recommendation of the American Diabetes Association for screening all women without prior known diabetes between 24 and 28^th^ gestational week^(8)^. The recommendation was based upon the inefficiency of the current history-based risk assessment method. However, the benefits of broad screening have not yet been established. A recent study by Koivunen et al.^(9)^ reported no benefit of broad screening on cesarean section rates and birthweight despite increased rates of GDM diagnosis, glucose-challenge test applications, and labor induction. Until the benefits of broad screening are established there is a need of a better risk assessment method.

Uric acid has been investigated as a possible risk factor for the development of GDM. Several researchers reported an association of uric acid levels with development of GDM^(10,11,12)^. The aim of the current study was to investigate the association of first trimester serum uric acid levels with development of GDM in a population of low-risk pregnant women.

## MATERIALS AND METHODS

This was a retrospective case-control study including pregnant women who were followed-up entirely at Ankara University Hospital between January 2012 and December 2014. Our antenatal follow-up program includes routine blood tests during the first trimester with biochemical panel and a two-step approach for screening of GDM, in accordance with the American Diabetes Association recommendations^(13)^. The two-step approach consists of a 50-g oral glucose challenge test (GCT) performed between the 24^th^ and 28^th^ weeks of gestation, followed by a 100-g oral GCT if the initial 50-g oral GCT serial glucose result is over 140 mg/dL. The results of the 100-g oral GCT were interpreted in accordance with the Carpenter and Coustan^(14)^ criteria for diagnosis of GDM. Uric acid levels were analyzed from serum samples obtained in the first trimester. Gestational ages were calculated from crown-rump length measurements in the first trimester^(15)^.

Pregnant women who had completed both first trimester biochemical panel and GDM screening were included for analysis. Women with prior diabetes, hypertension, chronic kidney disease, multiple pregnancy, chronic liver disease, gout arthritis or history of alcohol use were excluded from the analysis. The primary outcome of the study was the association of uric acid levels with occurrence of GDM. For the statistical analysis, the pregnant women were divided into three groups according to their respective GDM screening results. Women who took the 100-g oral GCT were divided into two groups, those whose results indicated GDM (GDM group) and those whose results had at least one abnormal or no abnormal results and did not meet the criteria for diagnosis of GDM (impaired glucose tolerance group). A maternal age-, gestational age-, and body mass index (BMI) matched control group was used to compare uric acid levels between groups.

To determine the size of the case and control groups, first trimester serum uric acid levels of a small group of healthy pregnant women without GDM were used. The mean uric acid level of this group was 3.72 mg/dL ±1.14. To detect a 0.5 mg/dL mean between-group difference in uric acid levels, 41 samples in each group was required for the study to have 80% or more power with a two-sided type I error rate of 0.05.

All statistical analyses were performed using SPSS version 21.0 (IBM Corporation, Armonk NYC, USA). Parameters with normal distribution are described in means with standard deviation. Parameters with non-normal distribution are described in median with minimum maximum values. Student’s t-test was used to compare continuous variables between independent groups. For each group, one-way ANOVA was used to test maternal age, pre-gestational BMI, and the gestational week serum samples as covariates to see if adjustment in a multivariable logistic regression model was necessary. Receiver-operating characteristics (ROC) curves were used to test the utility of first trimester serum uric acid levels for diagnosis of GDM. P values below 0.05 were considered statistically significant. This study was considered exempt from ethical approval by the Local Ethics Committee of Ankara University.

## RESULTS

A total of 4.812 pregnant women completed their antenatal follow-up in Ankara University Department of Obstetrics and Gynecology outpatient clinic between 2012 and 2014. Five hundred ten pregnant women were scheduled for a 100-g oral GCT because of a positive result of 50-g oral GCT. The results of the 100-g oral GCT revealed that 86 women had GDM and the remaining 410 women were diagnosed as having IGT. The prevalence of GDM was 1.7% in our study group. Twenty-six women in the GDM group and 66 women in the IGT group were excluded from the final analysis because they had co-morbidities (gestational hypertension, chronic liver or kidney diseaes), multiple gestations or missing first trimester serum uric acid levels. Two hundred two healthy age- and BMI-matched pregnant women were included as a control group. Baseline characteristics of the study groups can be found in Table 1.

In each separate group, one-way ANOVA was used to test for an association of BMI, maternal age, and gestational age serum samples measured for serum uric acid levels, which revealed no association of tested covariates with serum uric acid levels (p>0.05).

**Figure 1:**
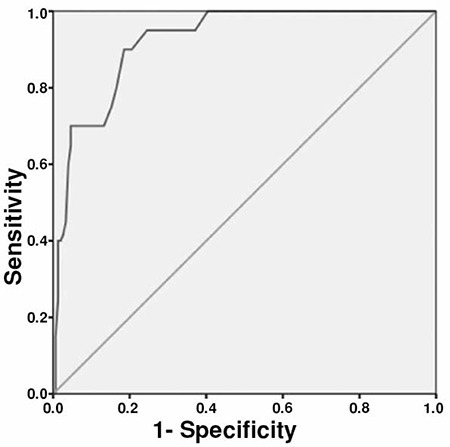
Receiver-operating characteristics curve for prediction of gestational diabetes mellitus with first trimester serum uric acid levels

Student’s t test revealed serum uric acid levels were significantly different between the groups with 5.94±0.97 mg/dL in the GDM group, 4.76±1.51 mg/dL in IGT group, and 3.76±1.07 mg/dL in the control group (p<0.001). ROC curve was obtained for the first trimester serum uric acid levels to detect GDM (Figure 1). The area under the curve was 0.92 [95% CI: (0.88-0.95)] with a diagnostic threshold of 3.95 mg/dL; first trimester serum uric acid levels had a sensitivity of 100% and specificity of 60% for the prediction of GDM.

## DISCUSSION

Uric acid is the final product of the oxidation step of purine catabolism and is an important marker for insulin resistance and the future development of metabolic syndrome. The prevalence of GDM is rising across the globe and the benefits of broad screening for GDM has not yet been proven^(9,16)^. Considering that the prevalence of GDM varies greatly between populations, a better risk assessment model could prevent unnecessary oral GCTs for screening of GDM, especially in populations such as ours where the prevalence of GDM is exceedingly low. In our study, we saw that first trimester uric acid levels had a linear association with the development of GDM and IGT. First trimester serum uric acid levels along with other parameters such as sex-hormone binding globulin, high-sensitive C-reactive protein, and adiponectin could be incorporated into a risk model to assess the need for oral GCT later in pregnancy^(17,18)^.

The strong points of our research are that our test sample was sufficiently powered and also demonstrated the diagnostic power of serum uric acid levels in a population of pregnant women with very low prevalence of GDM. Our study was retrospective in nature and had certain limitations that might have confounded our results, such as missing data in the study group and limited control over the study groups.

Our study adds to the body of literature about the association of serum uric acid levels with the development of GDM^(10,11,12,20)^. There is a conflicting study by Maged et al.^(21)^ which suggested no association, but their study was insufficiently powered to demonstrate a lack of difference between groups. Our study is different from the previous study with a GDM prevalence of 1.7%, which is much lower than other studies, and it is the first to report the association and predictive value of the test in a low-prevalence population. Further studies in this field should investigate the predictive value of uric acid levels combined with other biochemical tests in an effort to create a screening model.

## CONCLUSION

In summary, first trimester serum uric acid levels are associated with subsequent development of IGT and GDM. The test has good predictive value for the diagnosis of GDM and it can be used in a risk assessment model.

## Figures and Tables

**Table 1:**
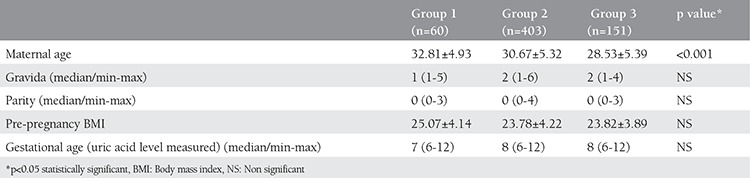
Maternal demographic characteristics
